# In Vitro Lethality of Fenbendazole to the Eyeworm *Oxyspirura petrowi*

**DOI:** 10.3390/ani14111659

**Published:** 2024-06-01

**Authors:** Jeremiah Leach, Hannah N. Suber, Emilynn Banks, Ashley Kaskocsak, Henry Valencia, Benjamin Hames, Regan Rivera, Sarah Colette, Ronald J. Kendall

**Affiliations:** Wildlife Toxicology Laboratory, Texas Tech University, 1234 Davis Dr., Lubbock, TX 79416, USA

**Keywords:** *Oxyspirura petrowi*, fenbendazole, lethality, in vitro, LC_50_, nematode, benzimidazole

## Abstract

**Simple Summary:**

There are growing concerns about wildlife and livestock interactions and the impacts of those interactions on the sustainability of livestock. One of those concerns is the spillover of wildlife pathogens, including helminths, into livestock. This concern will likely become realized as the demand for free-range animal products increases. One such helminth with spillover potential is the eyeworm *Oxyspirura petrowi*. This eyeworm is common in many wild birds, and particularly common in Northern bobwhite quail. Related helminths are already commonly found in poultry raised in free-range conditions in developing nations. The purpose of this research was to investigate the lethality of fenbendazole, a widely available drug for treating parasites, to these eyeworms. The lethality estimates were similar to estimates of lethality to other roundworm parasites. However, studies that have investigated concentrations in host blood following administration of the drug indicate that it does not stay in the system long enough to achieve elimination of the parasite after a single dose. This indicates that in order to effectively treat eyeworm, fenbendazole must be delivered in a repeated or continuous manner.

**Abstract:**

*Oxyspirura petrowi* is a heteroxenous nematode that infects the harderian gland and other ocular tissues in birds. High-intensity infections often cause damage to the infected tissues. Due to the nature of the infection sites, treatment of *O. petrowi* in these hosts can be difficult. Fenbendazole (FBZ) is a common anthelmintic used to treat birds for helminth infections; however, little information exists as to the efficacy of the drug on *O. petrowi* infections. The present study aims to estimate lethal concentrations of FBZ to *O. petrowi*. Adult *O. petrowi* were maintained in vitro and exposed to doses of 5, 50, 100, and 200 µM concentrations of FBZ and included both negative and vehicle controls. Exposure lasted 7.5 days and lethality was determined for each treatment. Negative and vehicle controls did not differ, and both had 75% survival at the end of the treatment period. The percentage survivorship in ascending order of concentration, corrected for the controls, was 66.67%, 44.44%, 33.33%, and 0%. LC_10_, LC_50_, and LC_90_ estimates were 7.5 ± 0.26, 49.1 ± 1.69, and 163.2 ± 5.63 µM, respectively. In the context of known pharmacokinetics of FBZ in birds, a single oral dose of FBZ can achieve exposure levels that are lethal to *O. petrowi*, but the drug does not stay in the system long enough. Thus, treatment of *O. petrowi* infections will require multiple oral doses over several days.

## 1. Introduction

Fenbendazole (FBZ), a member of the benzimidazole class of drugs, is a broad-spectrum anthelmintic approved for treating gastrointestinal helminth infections in various types of livestock. This anthelmintic is also used to treat helminth infections in people. The drug works by interacting with β-tubulin molecules and preventing the formation of microtubules in the cell [1]. This results in a collapse of cell structure and subsequent death of the target parasite [2]. Since exposure is often through ingestion, the consequence of benzimidazole action on nematode intestinal cells has been well studied and has been shown to impair digestion and excretion in *Haemonchus contortus*, a nematode that infects the rumen of many ungulates [3]. The effectiveness of FBZ as a nematicide and its relative safety in vertebrates has made FBZ an important anthelmintic [4]. 

*Oxyspirura petrowi* is a heteroxenous nematode commonly found to infect wild birds in North America [5,6,7,8,9]. This eyeworm can be highly prevalent in quail and passerines in endemic areas. Surveys in West Texas revealed an 89–100% prevalence and a mean abundance of 44 worms in wild quail [10,11,12,13]. These high-intensity infections in quail were also correlated with cell atrophy, eye inflammation, edema, and damage to the cornea and eye ducts [13,14]. *Oxyspirura petrowi* has also been found in songbirds at prevalences of 42.9%, 85.7%, and 100% in Northern cardinals (*Cardinalis cardinalis*), Northern mockingbirds (*Mimus polyglottus*), and Curve-billed thrashers (*Toxostoma curvirostre*), respectively [6]. The prevalence and abundance of *O. petrowi* in wild birds is so great that concern of spillover into domestic poultry in Michigan was expressed in 1935 [15]. These concerns will likely be realized with increasing demand for free-range poultry products as *Oxyspirura* spp. infections have already been found in poultry kept in free-ranging conditions [16,17,18].

The global distribution and epidemic potential of *O. petrowi* and congenerics make understanding control methods a priority, especially if pharmaceutical interventions become more relevant in their control. The goal of this research was to explore the pharmacodynamics of FBZ to *O. petrowi* and put it in the context of known pharmacokinetics. Knowing the concentrations at which anthelmintics cause lethality to *O. petrowi* is important and will allow for more effective control of *O. petrowi* populations in hosts and can inform future work of the in vivo efficacy of FBZ to *O. petrowi* and its congeners. The objective of this study is to assess the dose-response of *O. petrowi* to the anthelmintic FBZ via in vitro methods to quantify survivorship. FBZ was selected as the anthelmintic for this study because it is widely available and already approved in several nations for use in many domestic bird species. 

## 2. Methods

### 2.1. Chemical Source and Quality

FBZ was obtained from Sigma-Aldrich^®^ (Darmstadt, Germany) (≥98% purity, lot number MKBR9907V). Dimethyl sulfoxide (DMSO) was obtained from Fisher Chemical^®^ (Pittsburgh, Pennsylvania, USA) (ACS Grade, lot number 187334). Sodium phosphate monobasic (NaH_2_PO_4_) and potassium phosphate monobasic (KH_2_PO_4_) were both obtained from Sigma-Aldrich^®^ (Darmstadt, Germany), lot numbers BCBL2768V and SLBD9446V, respectively. Sodium chloride (NaCl) was obtained from Fisher Chemical^®^ (Pittsburgh, Pennsylvania, USA) (lot number 135570). NaH_2_PO_4_, KH_2_PO_4_, and NaCl were all ≥99% purity. Egg whites were obtained from a local market. 

### 2.2. Solution Preparation and Treatment Groups

Worms were split into a total of six groups for FBZ lethality testing. The groups were control, vehicle control, and four treatment groups. Each of the treatment and control solutions were made based on Dunham et al. [19]. The control solution consisted of physiological saline, as described in Corba et al. [20], mixed with egg white at a 1:1 ratio. The physiological saline was made using distilled water and sterilized by autoclave. The physiological saline for vehicle controls and all treatments was made using 3% DMSO. Vehicle controls and all treatments consisted of 50% drug solution and 50% egg white to obtain a final concentration of 1.5% DMSO for the vehicle control and 1.5% DMSO with 5 µM (1.5 ppm) concentration, 50 µM (15 ppm) concentration, 100 µM (30 ppm) concentration, and 200 µM (60 ppm) concentration. 

### 2.3. Eyeworm Collection

Eyeworms were collected from hunter-harvested wild *Colinus virginianus* from Fisher County, Texas. A researcher would follow along during the hunt and collect birds as they were harvested. The researcher would place the whole carcass in a resealable plastic bag and place the bag in a portable insulated box. The temperature of the box was maintained between 21 and 27 °C using Hothands™ single-use warmers (Kobayashi Healthcare, Dalton, Georgia, USA). The warmers were placed under a cloth towel in the bottom of the box and temperature was monitored using a digital thermometer. Temperature was checked at least once an hour and whenever the box was opened. The researcher would remove the heads of the birds and return them to the resealable bag and insulated box once the hunt was completed. The heads were then transported to the Wildlife Toxicology Laboratory at Texas Tech University where the eyes and associated tissues were inspected for eyeworms according to Dunham et al. [7]. Once removed from the host tissue, eyeworms were placed in 0.01 M PBS or control solution for assessment. Worms were considered suitable for use in this experiment if there was no visible damage and they demonstrated unprompted activity within 24 h of being collected. 

### 2.4. Experimental Design

The experimental protocol was carried out in six-well cell culture plates with six replicates. The wells of each plate were randomly assigned one of the six experimental conditions and 10 mL of the appropriate solution was added to the well. Four worms were placed in each well, beginning with well number 1, once all wells had the appropriate solution. Worms were maintained in a cell incubator at 40 C with 5% CO_2_. Worms were checked 12 h later, and then checked at 24-h intervals for a total of 7.5 days under 10× magnification and assessed as live or moribund. While not a definitive confirmation, the worms were deemed moribund if they failed to respond to gentle prodding with a metal probe. Percent mortality was assessed for each treatment and statistical analysis was completed using the R package drc in R Studio^®^ version 2023.06.0 Build 421 [21]. The model was fitted using the exponential decay function with the lower limit set at 0. Controls were then removed from the data, the model was run again, and the resulting model was used to estimate benchmark concentrations [22].

## 3. Results

**Figure 1:**
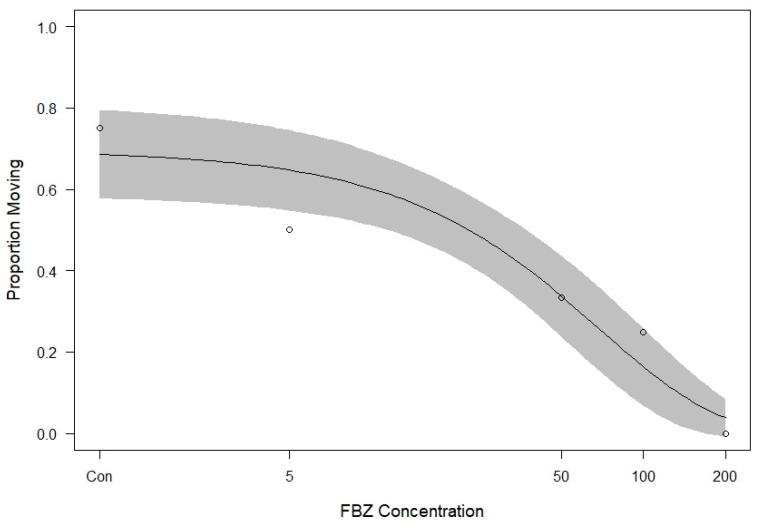
Proportion of worms found moribund when placed in a solution containing FBZ after 180 h with observed proportion moribund (circles) and 95% confidence interval (shaded area). Concentrations are in µM and control and vehicle control groups were pooled. Con = controls.

**Figure 2:**
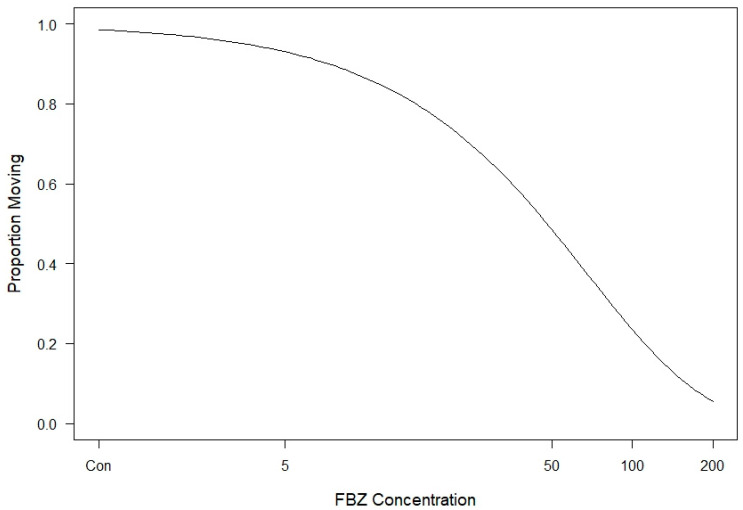
Dose–response curve of *O. petrowi* corrected for control groups after 7.5 days of exposure to FBZ. Con = controls.

A total of 29 *C. virginianus* heads were donated and examined for eyeworm infection. Prevalence of eyeworms was 86.2% and mean abundance was 11.7 worms/bird. In total, 144 worms were used to test the lethality of FBZ, with 24 worms in each treatment and control. Estimated parameters were statistically significant, with the plateau d = 0.695 (*p* < 0.0001) and k = 69.04 (*p* < 0.0001). Table 1 shows the survival of worms at each time point for the concentrations used in this study. The dose–response curve in relation to FBZ concentration at 7.5 days post-treatment with 95% confidence intervals is shown in Figure 1. The percent mortality relative to control and DMSO treatments are displayed in Table 2 and the mortality curve corrected for the control groups is shown in Figure 2. Estimates of LC_10_, LC_50_, and LC_90_ and their standard errors are 7.5 ± 0.26 µM (2.47 ± 0.079 ppm), 49.1 ± 1.69 µM (14.7 ± 0.51 ppm), and 163.2 ± 5.63 µM (48.65 ± 1.69 ppm), respectively. 

## 4. Discussion

This represents the first report of the in vitro lethality of FBZ on the eyeworm *O. petrowi*. The percentage of control worms surviving to the end of this experiment was similar to the percent surviving in other in vitro studies of *O. petrowi*. Dunham et al. [19] reported 75% survival after 10 days, reflected here in the survival of control and vehicle control worms after 7.5 days. Survivability of worms was reduced even at low concentrations of FBZ and all worms were dead after 7.5 days in a solution containing 200 µM FBZ. The effect of FBZ on *O. petrowi* reported here is similar to reports in other nematodes. In the free-living nematode *Caenorhabditis elegans*, a concentration of 100 µM was sufficient to achieve 100% mortality in an albendazole susceptible strain [23]. FBZ concentrations of about 6.7 µM were sufficient to reduce populations of *Pristionchus maupasi*, a soil nematode, by over 50% relative to controls [24]. Substantial reduction of the viability of *Trichinella spiralis*, an intestinal worm that is transmitted through the ingestion of raw or undercooked meat, was observed at concentrations of 1.88 µM albendazole solution, a benzimidazole-class anthelmintic that is more lethal to *C. elegans* than FBZ [23,25]. Concentrations of 500 µg/mL (1.67 mM) of FBZ, greater than eight times the maximum dose used in this study, were sufficient to obtain nearly 90% lethality in *Ascaridia galli* after 36 h [26]. Based on the results of this study, the lethality of FBZ to *O. petrowi* is within the range of lethality to other nematodes. 

Interpreting these results in the context of naturally infected hosts is difficult. Studies of the pharmacokinetics of FBZ in livestock show substantial diversity in the absorption and secretion rate of the anthelmintic. FBZ is absorbed more slowly and tends to have longer systemic residence than other benzimidazoles [27], further increasing the difficulty of interpreting these data in the context of naturally occurring infections. A comparative study of FBZ in Droughtmaster cattle (*Bos indicus* and *B. taurus* cross) and Swamp buffalo (*Bubalus bubalis*) found higher FBZ concentrations in blood plasma and greater retention time in cattle [28]. Beagle dogs given a dose of 20 mg/kg body weight had concentrations of FBZ in their plasma greater than 0.1 µg/mL for nearly 24 h, with an area under the curve (AUC) of 9.74 µg·h/mL [29]. Plasma concentrations in goats were similar to that of the Beagle, with max concentrations of 0.19 µg/mL and concentrations greater than 0.01 µg/mL for nearly 24 h [30]. The AUC in goats administered an oral dose of FBZ at 7.5 mg/kg body weight was 4.76 µg·h/mL [30]. Metabolism and excretion of FBZ and its metabolites was more similar between domestic chickens (*Gallus gallus domesticus*) and domestic ducks (*Anas platyrhyncos domesticus*) than chickens and domestic turkeys (*Meleagris gallopavo f. domesticus*) [31]. While information on the pharmacokinetics of FBZ in birds is lacking, it appears to be highly variable, even within birds of the same order. Despite the variability in the available pharmacokinetic data, blood plasma concentrations of FBZ are below the concentrations used in this study. However, the AUCs reported suggest that while the expected FBZ exposure in vivo should be high enough to have lethal effects on *O. petrowi*, the drug simply does not have a long enough residency time. This suggests that the use of anthelmintics to treat *O. petrowi* infections should consist of multiple or continuous doses over time and is consistent with in vivo studies of oxfendazole and albendazole in poultry [32]. However, while the benchmark concentrations reported here should be attainable in the blood plasma, only an in vivo study conducted with the target host can verify efficacy of FBZ against *O. petrowi*. It is also worth considering that one of the major metabolites of FBZ is oxfendazole. Thus, any worm exposed to FBZ is also being exposed to oxfendazole, which is a potent anthelmintic in its own right [33]. As the detrimental effects of heavy parasite burdens in wildlife are being realized, an increase in anthropogenic intervention is likely, including drug treatment. Future research studying the pharmacokinetics of FBZ in the wildlife hosts would be extremely beneficial, especially if used in conjunction with the results of this study. It would allow for a prediction of the efficacy of FBZ in treating wildlife species for *O. petrowi*, which in turn would allow for the implementation of more efficient management plans for the wildlife of concern. 

## 5. Conclusions

Epidemics and spillover events will become more common as the world continues to change. This will very likely include an increase in the detrimental effects of helminths on wildlife and helminths common to wildlife finding their way into livestock and poultry. Understanding the response of enzootic helminths to available anthelmintics will become ever more imperative to controlling outbreaks. Here we demonstrated the use of an in vitro assay for measuring the effects of a common anthelmintic on *O. petrowi*, a helminth that is likely to occur in free-range poultry and is common in wild birds. The benchmark doses reported here are similar to those of other in vitro studies with nematodes; however, in vivo studies must be conducted in order to determine effective treatment plans and efficacy of fenbendazole against *O. petrowi* and its congeners. In vivo studies using other benzimidazole class anthelmintics suggest single-dose treatment regimens using recommended doses may not be suitable for treating *O. petrowi*. Furthermore, pharmacokinetic information indicates the FBZ does not have an appropriate residual time in the host. Thus, in vivo studies and treatment plans should focus on the use of medicated feeds with continuous or near continuous access for at least 7 days. Another alternative would be the co-administration of cytochrome P450 inhibitors, which have been shown to increase the concentration of FBZ in the blood plasma and the residual time [29]. Lastly, FBZ may have a wide margin of safety, but it can be toxic to vertebrates and particularly so at high doses. Future work investigating the efficacy of FBZ against *O. petrowi* should consider this during research planning. There is clearly still work to be done and it is our hope that this work will stimulate further investigations into best practices for treating helminths found outside of the host gastrointestinal tract. 

## Figures and Tables

**Table 1:** Percent survivorship of *O. petrowi* at increasing concentrations of FBZ. Sample size is denoted after the treatment group with the letter n.

Treatment	12 h	36 h	60 h	84 h	108 h	132 h	156 h	180 h
Con (n = 24)	100.00%	100.00%	91.67%	95.83%	91.67%	79.17%	75.00%	75.00%
DMSO (n = 24)	100.00%	100.00%	95.83%	87.50%	87.50%	79.17%	66.67%	75.00%
5 µM (n = 24)	100.00%	100.00%	100.00%	87.50%	87.50%	66.67%	50.00%	50.00%
50 µM (n = 24)	87.50%	83.33%	79.17%	79.17%	75.00%	66.67%	41.67%	33.33%
100 µM (n = 24)	100.00%	100.00%	95.83%	95.83%	91.67%	66.67%	41.67%	25.00%
200 µM (n = 24)	95.83%	95.83%	91.67%	87.50%	70.83%	54.17%	8.33%	0.00%

**Table 2:** Percent survivorship of FBZ-exposed worms relative to controls and DMSO-exposed worms combined.

Dose	Percent Control
Control and DMSO	100
5 µM	66.67
50 µM	44.44
100 µM	33.33
200 µM	0

## Data Availability

Data are available upon reasonable request to the corresponding author.
